# Expanding the Substantial Interactome of NEMO Using Protein Microarrays

**DOI:** 10.1371/journal.pone.0008799

**Published:** 2010-01-20

**Authors:** Beau J. Fenner, Michael Scannell, Jochen H. M. Prehn

**Affiliations:** Centre for Human Proteomics and Department of Physiology and Medical Physics, Royal College of Surgeons in Ireland, Dublin, Ireland; CNRS - Université Aix-Marseille, France

## Abstract

Signal transduction by the NF-kappaB pathway is a key regulator of a host of cellular responses to extracellular and intracellular messages. The NEMO adaptor protein lies at the top of this pathway and serves as a molecular conduit, connecting signals transmitted from upstream sensors to the downstream NF-kappaB transcription factor and subsequent gene activation. The position of NEMO within this pathway makes it an attractive target from which to search for new proteins that link NF-kappaB signaling to additional pathways and upstream effectors. In this work, we have used protein microarrays to identify novel NEMO interactors. A total of 112 protein interactors were identified, with the most statistically significant hit being the canonical NEMO interactor IKKbeta, with IKKalpha also being identified. Of the novel interactors, more than 30% were kinases, while at least 25% were involved in signal transduction. Binding of NEMO to several interactors, including CALB1, CDK2, SAG, SENP2 and SYT1, was confirmed using GST pulldown assays and coimmunoprecipitation, validating the initial screening approach. Overexpression of CALB1, CDK2 and SAG was found to stimulate transcriptional activation by NF-kappaB, while SYT1 overexpression repressed TNFalpha-dependent NF-kappaB transcriptional activation in human embryonic kidney cells. Corresponding with this finding, RNA silencing of CDK2, SAG and SENP2 reduced NF-kappaB transcriptional activation, supporting a positive role for these proteins in the NF-kappaB pathway. The identification of a host of new NEMO interactors opens up new research opportunities to improve understanding of this essential cell signaling pathway.

## Introduction

Nuclear factor κ-light chain enhancer of activated B cells (NF-κB) is a global transcriptional regulator found in most animal cells and is involved in responses to a wide variety of stimuli including cytokines such as TNFα, pathogens, free radicals, hypoxia, UV irradiation and other stresses [1], [2], [3], [4]. NEMO, the NF-κB essential modulator, was originally described as being required for activation of the NF-κB pathway in response to such stresses [5]. Subsequent work revealed that the 48 kDa NEMO protein serves as an adaptor that links stimulation of upstream signaling components such as the membrane-bound TNFα and interleukin-1 receptors to the activation of IκB kinase proteins, IKKα and IKKβ [6], [7]. Once activated, the IKK proteins phosphorylate IκB, targeting it for proteosomal degradation and liberating the NF-κB transcription factor. Free NF-κB then enters the nucleus and activates transcription of its target genes.

**Figure 1:**
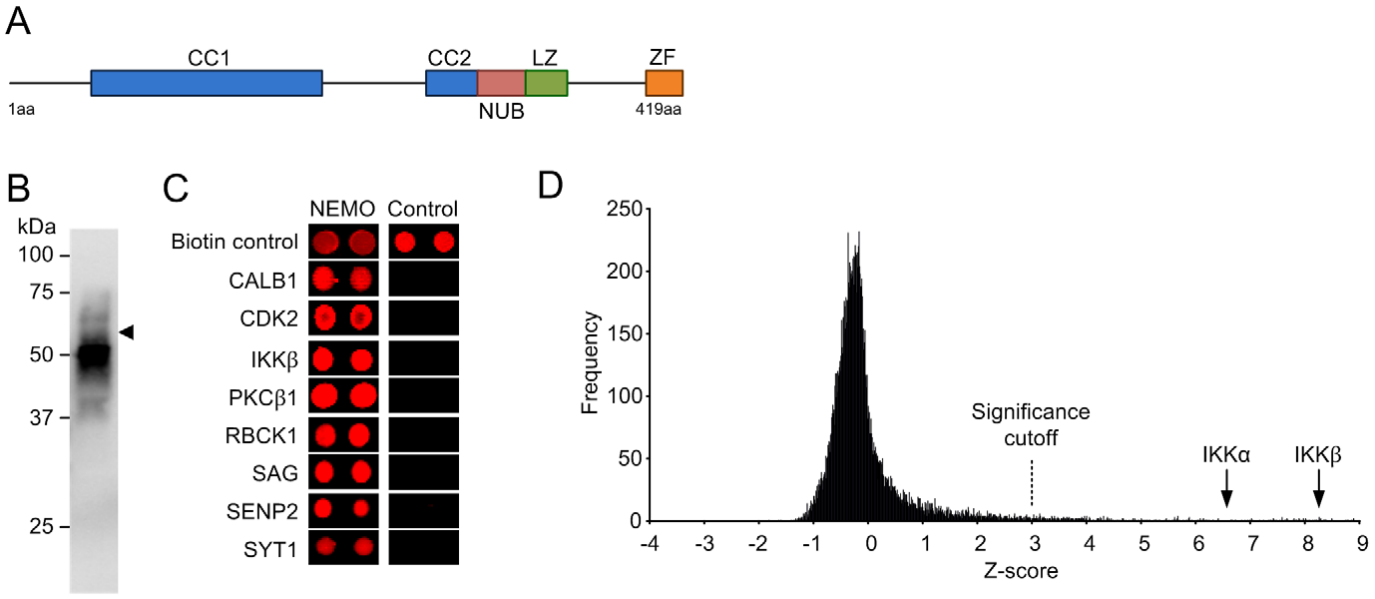
Probing of the human protein microarray with biotinylated recombinant NEMO. (A) Domain structure of the human NEMO protein, showing the two coiled coil domains (CC1 and CC2), the NEMO ubiquitin binding domain (NUB), leucine zipper (LZ) and zinc finger (ZF). (B) Immunoblot detection of biotinylated NEMO following purification of GST-NEMO, cleavage of the GST tag and biotinylation. Biotinylated NEMO was detected with streptavidin-alkaline phosphatase conjugate. (C) Example hits obtained from the array, compared to the same spot positions on negative control array. (D) Frequency histogram for the NEMO-probed protein microarray showing the range of Z-scores obtained. Protein interactors with a *Z*-score greater than three (Z>3; P<0.002) were deemed significant. Scores obtained for the canonical NEMO interactors IKKalpha (Z* = *6.52) and IKKbeta (Z = 8.41) are shown for reference. Scores were calculated using Invitrogen Protoarray Prospector version 5.1 software. See Table 1 for gene descriptions.

In the case of the TNFα receptor, cytoplasmic NEMO is recruited to the stimulated TNFα receptor complex by binding to K63-linked polyubiquitin chains that are conjugated to RIP1 upon receptor activation [8], [9], [10]. Binding to the polyubiquitin chains is mediated by the NEMO ubiquitin binding domain [11; see Figure 1], which binds multiple forms of polyubiquitin but prefers K63- over K48-linked polyubiquitin [12].

While the specific function of NEMO in relation to TNFα receptor stimulation is its most well characterized role to date, a growing body of work points to a far more variable role. In response to genotoxic agents that induce genomic DNA strand breaks, NEMO acts independently of IKKα and IKKβ by entering the nucleus and associating with ATM [13], a process that depends on NEMO SUMOylation [14]. ATM promotes NEMO phosphorylation and, by mechanisms that remain unclear, NEMO is deSUMOylated, ubiquitinated and leaves the nucleus to activate the canonical IKK-dependent NF-κB pathway [13], [15]. Thus, at the very least, NEMO is predicted to have a substantial interactome of both nuclear and cytoplasmic proteins. Indeed, NEMO interactors identified to date include proteins involved in apoptosis induction, heat shock response, neuronal function and other cytokine signaling pathways [16], [17].

In the current work, we have used functional proteomics to identify and characterize novel NEMO interactors. Using human protein microarrays, we identified 112 NEMO binding proteins, including a large number of signaling kinases and proteins related to development and the cell cycle. Validation of a subset of the interactors indicated that the screen did indeed yield authentic NEMO binders, several of which were able to influence the activity of the NF-κB signaling pathway.

## Results

### Using Protein Arrays to Identify NEMO Interacting Proteins

Identification of NEMO binding proteins was initially performed by screening *E. coli* colony macroarrays containing more than 30,000 recombinant human proteins (8,300 non-redundant proteins) with full-length recombinant GST-NEMO. We previously used these macroarrays in our laboratory to identify polyubiquitin binding proteins [12]. A screen of the macroarrays that used GST-NEMO as a probe consistently revealed binding to polyubiquitin and TANK (data not shown), both of which are known NEMO interaction partners [8], [18]. The canonical NEMO interactor IKKβ was present on the macroarray but no interaction was detected with the NEMO probe. Previous work has indicated that IKKβ can indeed be expressed as a soluble protein in *E. coli* [19], though we were consistently unable to show binding between NEMO and this or any other protein on the macroarray other than polyubiquitin and TANK.

To overcome the apparent limitations of the *E. coli* colony macroarrays, we opted to use a human protein microarray (Protoarray, Invitrogen) consisting of approximately 8,400 unique human proteins expressed as GST or His_6_ fusions in an insect cell/baculovirus expression system, with proteins being individually purified and spotted onto nitrocellulose-coated glass slides. Following purification and biotinylation of tag-free human NEMO (Figure 1B) we applied the protein to the slides and detected bound protein using streptavidin/Alexa Fluor-647. Biotinylated GST was applied to a separate microarray as a negative control. Analysis of the scanned NEMO array indicated that the biotin controls were successful, with numerous positive hits being obtained (Figure 1C and 1D).

### Recombinant NEMO Has a Substantial Interactome *In Vitro*

Statistically significant interactors were identified by measuring the mean spot intensity for the microarray sectors and using a Z-score of three (P = 0.002) as a cutoff value (Figure 1D). Surprisingly, the GST control protein also bound a substantial number of proteins on the array (data not shown), which were considered as nonspecific interactors and where eliminated from the NEMO interactor list where overlap occurred. This subtraction reduced the number of significant interactors from 200 to 112, with a mean Z-score of 4.33±1.36 (P<0.001), shown in Table 1 and Figure 1D. Among the final list, the majority of putative NEMO interactors appeared to be completely novel, as only five proteins, namely CALM2, IKKα, IKKβ, MCM7 and TBK1 have previously been shown to interact directly with NEMO [17]. Neither UbC nor TANK was present on the microarrays so it was not possible to compare these results with those mentioned above for the *E. coli* colony macroarrays.

**Table 1:** Candidate NEMO interactors identified by protein microarray screening with full-length NEMO protein.

Gene	Gene Product	NCBI Accession	Z-Score
Calcium binding			
CETN3	Centrin EF-hand protein 3	BC005383	6.10
SYT1	Synaptotagmin I	NM_005639	5.95
CPNE2	Copine II	NM_152727	5.86
HPCAL1	Hippocalcin-like 1	NM_002149	4.20
MYL5	Myosin light chain 5, regulatory	NM_002477	3.83
CALB1	Calbindin D28K	NM_004929	3.61
TTYH2	Tweety homolog 2	BC004233	3.42
TPT1	Tumor protein, translationally-controlled 1	BC022436	3.40
DNA binding			
H1F0	H1 histone family, member 0	BC029046	4.40
MCM7^b^	MCM7 minichromosome maintenance deficient 7	BC009398	3.38
CNOT7	CCR4-NOT transcription complex, subunit 7	BC060852	3.21
GTPase			
CENTA2	Centaurin, alpha 2	BC033758	4.06
TBC1D7	TBC1 domain family, member 7	NM_016495	3.13
GNGT1	G protein, γ-transducing activity polypeptide 1	NM_021955	3.04
SEPT9	Septin 9	BC054004	3.03
Kinase			
IKBKB^b^	Inhibitor of kappa light polypeptide gene enhancer in B-cells, kinase beta	NM_001556	8.41
PRKCB1	Protein kinase C type beta I	X06318	8.33
TBK1^b^	Tank binding kinase 1	NM_013254	7.37
PRKCB2	Protein kinase C type beta II	X07109	6.83
IKBKA^b^	Inhibitor of kappa light polypeptide gene enhancer in B-cells, kinase alpha	NM_001278	6.52
LCK	Leukocyte-specific protein tyrosine kinase	M36881	5.97
CDK2	Cyclin-dependent kinase 2	NM_001798	5.34
FLT3	FMS-related tyrosine kinase 3	NM_004119	4.36
SRC2	Gardner-Rasheed feline sarcoma viral (v-fgr) oncogene homolog	NM_005248	4.32
FLT3 D835Y	FMS-related tyrosine kinase 3 (D835Y mutant)	NM_004119	4.24
STK25	Serine/threonine kinase 25	NM_006374	4.21
PIM2	Pediatric index of mortality 2	NM_006875	4.13
EPHA4	Ephrin receptor A4	NM_004438	4.10
TEK	TEK tyrosine kinase	NM_000459	4.09
JAK2	Janus kinase 2	NM_004972	3.96
TEC	Tyrosine kinase expressed in hepatocellular carcinoma	NM_003215	3.92
FLT4	FMS-related tyrosine kinase 4	NM_182925	3.89
RPS6KB2	Ribosomal S6 kinase	NM_003952	3.86
GRK4	G protein-coupled receptor kinase 4	NM_182982	3.84
PRKCN	Protein kinase D3	NM_005813	3.66
MAP3K2	Mitogen-activated protein kinase kinase kinase 2	NM_006609	3.66
AKT1	v-akt murine thymoma viral oncogene homolog 1	BC000479	3.65
SGK1	Serum/glucocorticoid regulated kinase 1	NM_005627	3.62
ITK	IL2-inducible T-cell kinase	NM_005546	3.54
TYRO3	TYRO3 protein tyrosine kinase	NM_006293	3.49
MERTK	c-Mer proto-oncogene tyrosine kinase	NM_006343	3.43
ALK	Anaplastic lymphoma receptor tyrosine kinase	NM_004304	3.42
JAK3	Janus kinase 3	NM_000215	3.36
RET	RET proto-oncogene	NM_020975	3.26
ROR2	Receptor tyrosine kinase-like orphan receptor 2	NM_004560	3.21
SRPK1	SFRS protein kinase 1	NM_003137	3.11
PCK1	Phosphoenolpyruvate carboxykinase 1	NM_002591	3.08
ROS1	c-Ros oncogene 1	NM_002944	3.08
DAPK1	Death-associated protein kinase 1	NM_004938	3.01
Protein binding			
CUEDC1	CUE domain containing 1	NM_017949	7.38
RNF7	Ring finger protein 7 (sensitive to apoptosis gene, SAG)	NM_014245	6.08
RBCK1	RanBP-type and C3HC4-type zinc finger containing 1	BC015219	5.25
PSMA3	Proteasome (prosome, macropain) subunit, alpha type, 3	NM_002788	5.19
WDR5	WD repeat domain 5, transcript variant 1	NM_017588	4.85
LZIC	Leucine zipper and CTNNBIP1 domain containing	NM_032368	4.41
ARL6IP4	ADP-ribosylation-like factor 6 interacting protein 4	NM_016638	4.25
PDCL	Phosducin-like	BC017227	4.04
PFDN5	Prefoldin 5, transcript variant 1	NM_002624	3.78
LPXN	Leupaxin	NM_004811	3.67
C9ORF32	Chromosome 9 open reading frame 32	BC001396	3.63
GADD45G	Growth arrest and DNA-damage-inducible, gamma	NM_006705	3.63
LUC7L2	LUC7-like 2	BC042625	3.62
GADD45GIP1	Growth arrest and DNA-damage-inducible, gamma interacting protein 1	BC013039	3.35
PHF7	PHD finger protein 7	NM_016483	3.27
SCLT1	Sodium channel and clathrin linker 1	BC064428	3.23
NAP1L5	Nucleosome assembly protein 1-like 5	NM_153757	3.20
TRIM41	Tripartite motif-containing 41	BC009762	3.20
APRT	Adenine phosphoribosyltransferase	NM_000485	3.19
KRT8	Keratin 8	BC008200	3.11
C19ORF57	Chromosome 19 open reading frame 57	BC012945	3.03
NECAB3	N-terminal EF-hand calcium binding protein 3	BC047673	3.03
MCM10	Minichromosome maintenance complex component 10	BC009108	3.01
RPAP3	RNA polymerase II associated protein 3	BC056415	3.00
RNA binding			
RBM34	RNA binding motif protein 34	NM_015014	5.64
NOLA2	Nucleolar protein family A, member 2	NM_017838	5.41
EIF1AX	Eukaryotic translation initiation factor 1A, X-linked	NM_001412	4.30
DDX19B	DEAD box polypeptide 19B, transcript variant 1	NM_007242	4.18
RBM8A	RNA binding motif protein 8A	NM_005105	3.92
ARL6IP4	ADP-ribosylation-like factor 6 interacting protein 4	BC015569	3.48
RPS12	Ribosomal protein S12	NM_001016	3.36
RPL41	Ribosomal protein L41	NM_021104	3.33
SIP1	Survival of motor neuron protein interacting protein 1, transcript variant alpha	NM_003616	3.31
Transcription factor			
MLLT6	Myeloid/lymphoid or mixed-lineage leukemia; translocated to, 6	BC064612	7.68
TCEANC	TFS2-M domain-containing protein 1	NM_152634	7.28
TCP10L	t-complex 10 (mouse)-like	NM_144659	6.82
TCP11L1	t-complex 11 (mouse)-like 1	NM_018393	5.68
LMCD1	LIM and cysteine-rich domains 1	NM_014583	4.63
NRBF2	Nuclear receptor binding factor 2	BC011707	4.08
Other/unknown			
FAM128A	Family with sequence similarity 128, member A	BC018206	7.32
TSLP	Thymic stromal lymphopoietin, transcript variant 2	NM_138551	6.61
DNAJC8	DnaJ (Hsp40) homolog, subfamily C, member 8	NM_014280	5.84
FRMD8	FERM domain containing 8	BC051695	5.71
C19ORF12	Chromosome 19 open reading frame 12	NM_031448	5.51
TCP11	t-complex 11 (mouse)	NM_018679	5.29
HBZ	Hemoglobin, zeta	NM_005332	4.59
FLJ11184	Hypothetical protein FLJ11184	BC011842	4.39
VAMP3	Vesicle-associated membrane protein 3 (cellubrevin)	NM_004781	4.07
KIR3DX1	Killer cell immunoglobulin-like receptor, three domains, X1	BC033195	4.02
ARGLU1	Arginine and glutamate rich 1	BC050434	3.99
GYG2	Glycogenin 2	BC023152	3.91
PNMAL1	PNMA-like 1	BC051688	3.91
LGALS2	Lectin, galactoside-binding, soluble, 2 (galectin 2)	NM_006498	3.72
ODAM	Odontogenic, ameloblast associated	NM_017855	3.54
AMMECR1L	AMME chromosomal region gene 1-like	NM_031445	3.53
PLEKHJ1	Pleckstrin homology domain containing, family J member 1	NM_018049	3.42
AVPI1	Arginine vasopressin-induced 1	NM_021732	3.33
SENP2	SUMO specific peptidase 2	NM_021627	3.32
ACBD6	Acyl-Coenzyme A binding domain containing 6	NM_032360	3.14
KIAA1598	Shootin-1	NM_001127211	3.05
NRARP	NOTCH-regulated ankyrin repeat protein	NM_001004354	3.04
ARPP-19	Cyclic AMP phosphoprotein, 19 kDa	NM_006628	3.01

*aThe Z-score indicates the how far the average spot intensity for a particular putative interactor fell from the mean of the relevant protein microarray sector spot intensities, measured in standard deviations. A Z-score of greater than four standard deviations (P = 0.002) was deemed significant.*

*bThese proteins are known to interact directly with NEMO, based on the results of tandem affinity purification experiments [17].*

**Figure 2:**
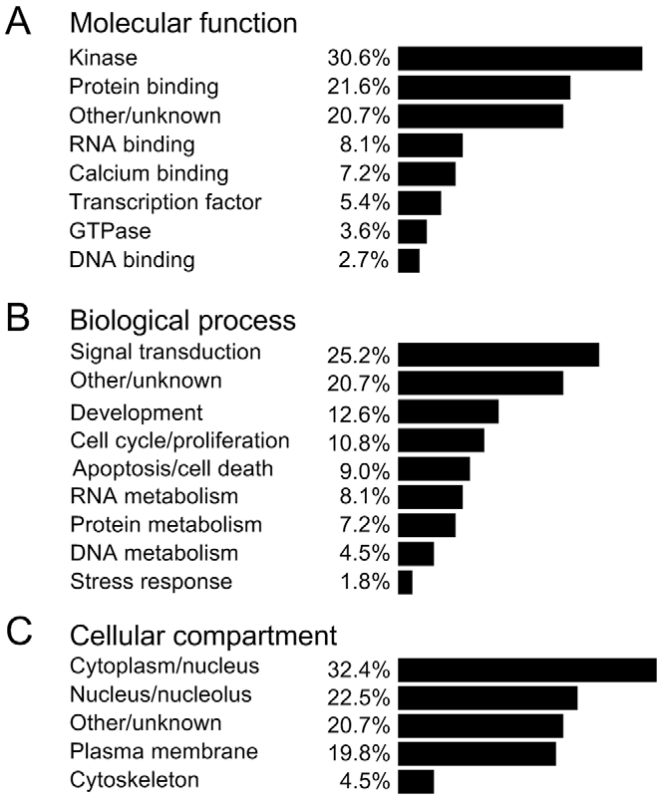
Cytoplasmic and nuclear signaling kinases dominate the NEMO interactome. Gene ontologies were determined for each of the NEMO interactors and the results for each of the three standard ontological categories plotted as percentages. Genes belonging to more than one category were assigned to the category for which the gene has been best characterized.

An ontological analysis of the putative interactors revealed that protein kinases were dramatically overrepresented in the dataset, comprising at least 30% of all identified interactors compared to the protein microarray used which only had 5% kinases (Figure 2A). That said, of all kinases present on the array, the kinases observed to significantly bind NEMO represented only 8% of the total number of arrayed kinases, suggesting that the interactions did not rely simply on a generic kinase motif. Importantly, the identified NEMO-interacting kinases included the canonical NEMO binding partners IKKα and IKKβ (Figure 1C, Table 1), with IKKβ having the highest apparent affinity for NEMO of all identified protein interactors. Given the canonical role of IKKβ as part of the IKK complex, this finding was considered a strong validation of the specificity and usefulness of the screen. Other kinases with a high apparent affinity for NEMO included PKCβ1, PKCβ2, LCK, CDK2, FLT3 and another known NEMO binding partner, TBK1 (Table 1).

Further ontological analysis revealed that signal transduction proteins formed the largest biological process category, with 25% of the interactome, while 11% and 13% of interactors are involved in cell cycle and development, respectively (Figure 2B). Despite the well-characterized role of the NF-κB pathway in regulating cell survival, only 9% of interactors are involved in apoptosis and cell death-related processes, while 2% are related to stress responses. As expected, the majority of identified NEMO interactors localize to the cytoplasm and/or nuclear compartments (Figure 2C), consistent with the known subcellular distribution of NEMO.

NEMO also interacted with the E3 ubiquitin ligases SAG and RBCK1, which were previously reported to promote ubiquitination of NF-κB signaling components IκBα and TAB2/3, respectively [20], [21]. GST-NEMO also interacted with SYT1, a major neuronal synaptic calcium sensor [22]. NEMO was previously shown to interact with an extended variant of this protein, termed E-SYT1 [17].

### NEMO Binds to Putative Interactors Expressed in Mammalian Cells

Based on our analysis of the list of putative NEMO interactors, we selected five proteins of particular interest to validate the microarray screen using GST pulldown and coimmunoprecipitation assays. Proteins were chosen based on their previously described roles related to NF-κB signaling. Calbindin D28K (CALB1) is 28 kDa neuronal calcium binding protein that is transcriptionally regulated by NF-κB following stimulation by neurotrophic growth factor [23]. Cyclin-dependent protein kinase 2 (CDK2) associates with NF-κB during the G1/S phase transition of the cell cycle [24], while sensitive to apoptosis gene 1 (SAG) is an E3 ubiquitin ligase that appears to target IκBα for degradation during the G1/S phase transition and in carcinogenesis [20], [25]. The SUMO1-specific peptidase 2 (SENP2) was chosen due to the role of SUMOylation in regulating the cellular localization of NEMO [14], [26]. Finally, synaptotagmin 1 (SYT1) was chosen due to a previous finding indicating that NEMO can bind to an extended SYT1 variant, E-SYT1 [17] and to the essential role of synaptotagmin 1 in synaptic Ca^2+^ release [27], [28], [29].

**Figure 3:**
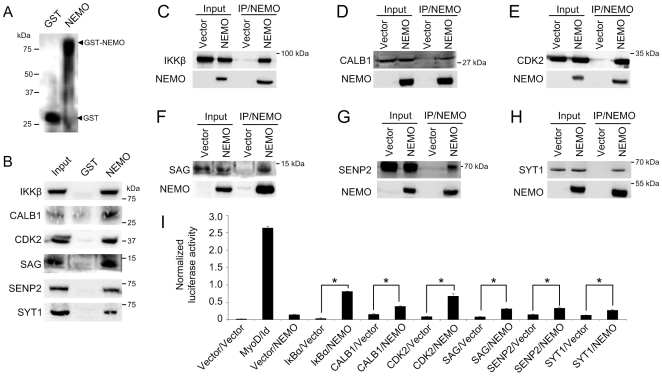
Putative interactors bind to NEMO in GST pulldown, coimmunoprecipitation and mammalian two-hybrid assays. (A) Immunoblot analysis of GST and GST-NEMO proteins used as control and bait for the pulldown assay. Proteins were detected using anti-GST/HRP conjugate following SDS-PAGE and membrane transfer. (B) Results of GST pulldown assays showing binding of NEMO to putative interactors identified by protein array screening. Each of the interactors and IKKbeta, a known NEMO binder, were overexpressed in transiently transfected HEK-293T cells and the resulting lysates applied to immobilized GST or GST-NEMO. Following incubation and washing, the samples were resolved by SDS-PAGE and the proteins detected using appropriate antibodies. Input lanes were loaded with 5–10% of HEK-293T lysates to confirm protein expression. The size of relevant protein markers is shown beside the blot image. (C–H) Coimmunoprecipitation assays between NEMO and putative binders in HEK-293T cells. Plasmids encoding Xpress-tagged NEMO or the empty parent vector and tagged putative binders were used to transfect HEK-293T cells and the resulting cell lysates used for coimmunoprecipitation assays. For each putative binder, immunoblots are shown for detection of the binder using a tag- or protein-specific antibody, and for detection of Xpress-tagged NEMO. For IKKbeta and each of the five putative interactors, substantial coimmunoprecipitation occurred only in the presence immunoprecipitated NEMO. Input lanes contained 5–10% of the precleared input volume used prior to addition of anti-Xpress antibody. Binding and washing steps were performed in the presence of 0.5% NP-40 for all proteins except SAG, where 0.1% NP-40 was used. (I) NEMO interacts with CALB1, CDK2, SAG, SENP2 and SYT1 in a mammalian two-hybrid system. Empty two-hybrid vectors were cotransfected as a negative control. The MyoD/Id and NEMO/IkappaBalpha protein pairs were used as positive controls, while putative interaction partners cotransfected with empty complementing vector were used as negative controls. For each pair tested, a significant increase (n = 6; two-tailed T test; P≤0.05) in luciferase activity was obtained in partner/NEMO experiments compared to partner/vector experiments (indicated by asterisks).

Thus, HEK-293T cells were transfected with plasmids expressing CALB1, CDK2, SAG, SENP2 or SYT1. Additionally, an IKKβ expression vector was transfected as a positive control. Using GST or GST-NEMO as bait proteins (Figure 3A), we found that each of the five interactors was able to specifically bind GST-NEMO, while little or no binding was observed with the GST control (Figure 3B). These data suggested that the observed array binding between NEMO and the interactors was indeed specific.

We next determined whether NEMO, when coexpressed with each of the five interactors, was able to form a complex *in vivo* using coimmunoprecipitation assays. Again, HEK-293T cells were transfected with expression vectors for NEMO and the five interactors, with IKKβ or the parent vector serving as positive and negative controls, respectively. In mammalian cells the parent vector, pcDNA4-HisMaxA, expresses a 5.6 kDa irrelevant protein in place of NEMO. As expected, IKKβ immunoprecipitated only in the presence of NEMO (Figure 3C). Similarly, each of the five novel NEMO interactors also exhibited NEMO-specific immunoprecipitation (Figure 3D–H), indicating that these proteins are can form a complex with NEMO *in vivo*. In the case of SAG, coimmunoprecipitation was observed in the presence of 0.1% NP-40 detergent, but not with 0.5% NP-40, while the remaining four proteins coimmunoprecipitated in the presence of 0.5% NP-40.

Finally, we used a two-hybrid system to validate the interactions between NEMO and the five interactors. A control experiment using two-hybrid luciferase vectors expressing NEMO and IκBα, a known NEMO interactor [17], showed a clear two-hybrid interaction between these two proteins when compared with the corresponding negative control (Figure 3I). Similarly, significant interactions were observed for each of the remaining NEMO interactors compared to their negative controls, with CDK2 appearing to have the highest affinity for NEMO on the basis of luciferase activity. A comparison between results obtained using the two-hybrid method and Z scores obtained from the original array screen did not reveal any obvious trend towards a high Z score and a strong two-hybrid signal, suggesting that array-based interactions do not correlate well to results obtained in a cellular environment.

### NEMO Interactors Influence NF-κB Transcriptional Activation

**Figure 4:**
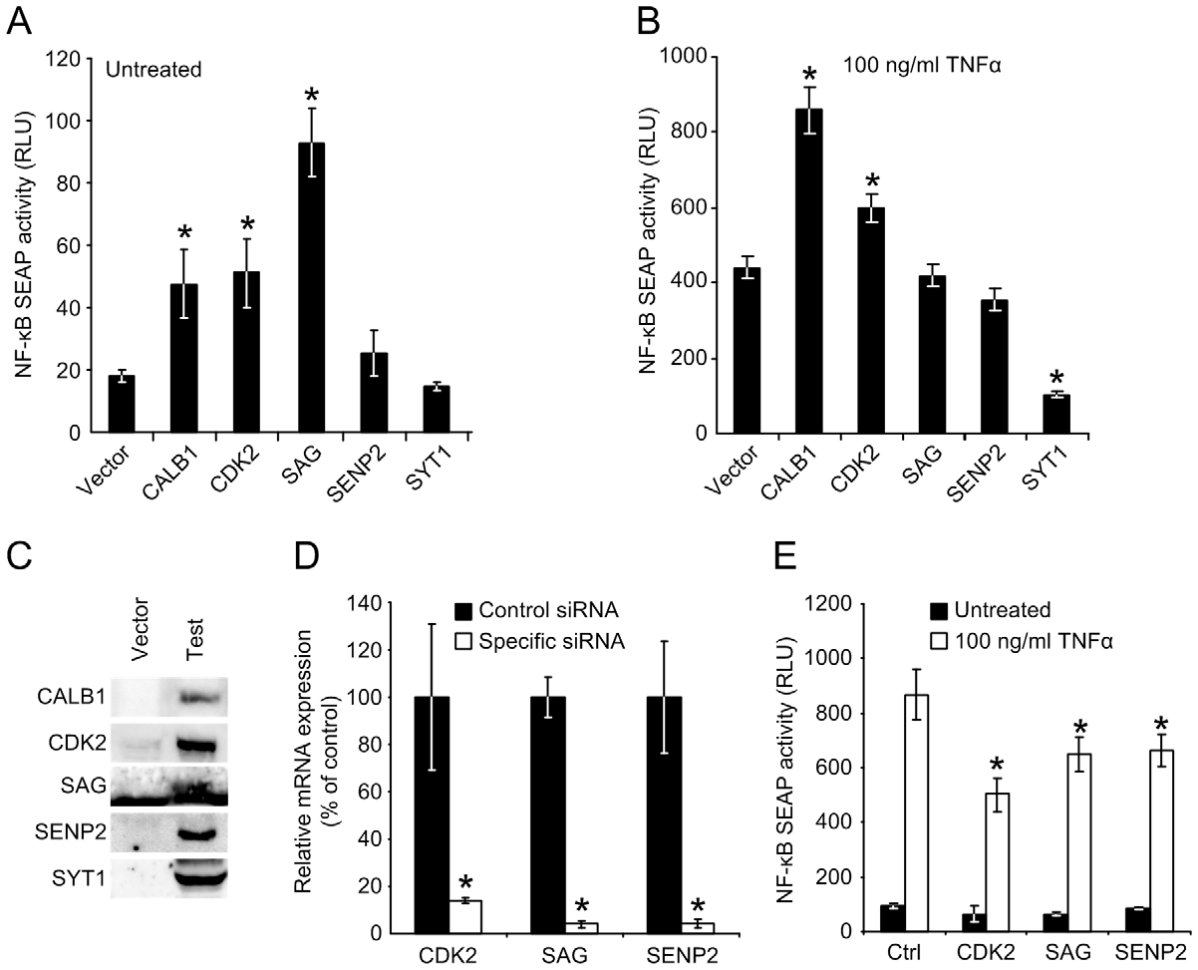
NEMO interactors influence the transcriptional activation activity NF-kappaB. (A) Each of the five NEMO interactors was overexpressed in untreated HEK-293T cells and their effect on NF-kappaB transcriptional activation measured by a reporter assay. CALB1, CDK2 and SAG significantly increased reporter activity, indicated by asterisks (n = 4; two-tailed T test; P≤0.05), while other genes had no effect compared to the control vector transfection. Reporter activity is given in relative light units (RLU). (B) CALB1 and CDK2 overexpressed increases NF-kappaB activity in TNFalpha treated cells, while SYT1 overexpression significantly represses activity (n = 4; two-tailed T test; P≤0.05). (C) Confirmation of protein expression following transfection of HEK-293T cells by immunoblot detection of native or epitope-tagged NEMO interactors. Little or no protein expression was detected in the control vector transfected cells. (D) Knockdown of CDK2, SAG and SENP2 in HEK-293T cells mediated by siRNA transfection. RNA levels at the time of NF-kappaB assays were measured by RT-qPCR and are displayed as percentage mRNA remaining after knockdown compared to the amounts present in control siRNA-treated cells. Significant knockdown was seen for both of the genes, as indicated by the asterisks (n = 3; two-tailed T test; P≤0.05). (E) mRNA knockdown of CDK2, SAG and SENP2 reduces NF-kappaB transcriptional activation in TNFalpha stimulated HEK-293T cells, but does not impact upon basal NF-kappaB activity in untreated cells (n = 4; two-tailed T test; P≤0.05).

To gain an understanding of how the identified interactors might contribute to the NF-κB pathway, we cotransfected HEK-293T cells with a secreted alkaline phosphatase (SEAP) NF-κB transcriptional reporter plasmid and expression plasmids for the five interactors. SEAP activity was then determined for cotransfected cells in the presence or absence of TNFα, a potent stimulator of NF-κB-dependent transcription. In the absence of TNFα we found that CALB1, CDK2 and SAG all significantly induced reporter activity (Figure 4A). After TNFα was added, we again found that CALB1 and CDK2 overexpression increased expression of the reporter above normal levels, while SYT1 substantially reduced induction of the reporter (Figure 4B). We did not observe any significant influence of SENP2 overexpression on reporter activity under these experimental conditions.

We next determined if transient siRNA-mediated mRNA knockdown of the interactors led to any change in reporter activity. For these experiments we chose to look at only CDK2, SAG and SENP2 as these were expressed at readily detectable levels in HEK-293T cells (data not shown), making mRNA knockdown feasible. Cotransfection of the reporter plasmid with control or specific siRNAs against these three putative NEMO interactors led to a highly effective siRNA knockdown of more than 80% by specific siRNAs compared to control siRNAs, based on the results of RT-qPCR using gene-specific primers (Figure 4D). Assays of reporter gene activity following gene knockdown yielded modest but significant reductions of NF-κB transcriptional activation for all three knockdowns when cells were treated with TNFα, though no significant change was observed between control and specific siRNA treatments in the absence of this stimulator. Thus, at endogenous levels, CDK2, SAG and SENP2 can be considered as positive effectors of the TNFα-dependent NF-κB pathway.

## Discussion

Signal transduction by the NF-κB pathway is typically considered as a “first response” mechanism as target gene activation by the NF-κB transcription factor occurs rapidly after stimulation by a wide variety of cellular stimuli. The position of NEMO within this pathway would therefore necessitate its ability to communicate with a large diversity of effectors to respond to these different stimuli. In this study, we have uncovered an exceptionally large number of NEMO interactors, most of which are novel and not previously known to communicate with the canonical NF-κB pathway.

While it is clear that many of the NEMO interactors identified here, including IKKα and IKKβ, are physiologically relevant, we concede that a certain number of the array proteins may not interact with NEMO *in vivo*. This could be due an inaccessible subcellular localization, inappropriate temporal expression patterns between NEMO and the interactors, or an overabundance of NEMO and the interactors used during array screening [30]. Surprisingly, we also noted that several of the false positive hits obtained on both the control and NEMO arrays have been reported elsewhere to specifically bind other protein baits [31], [32], [33]. While the interactions reported in those works may well be authentic, the recurrence of a number of protein hits during ProtoArray screens with other protein baits in our laboratory suggests that certain arrayed proteins become artificially promiscuous when arrayed.

Among the novel NEMO interactors identified, calbindin D28K and synaptotagmin 1 have well characterized neuronal functions. Calbindin D28K is commonly described as a neuronal calcium buffer that prevents the accumulation of toxic levels of calcium via four calcium binding domains [34], [35]. In addition to this buffering role, however, calbindin D28K also appears to function as a calcium sensor by interacting with downstream effector proteins [34]. Neuronal calcium-sensing roles are also played by three other novel NEMO interactors identified here and elsewhere, namely calmodulin [17], hippocalcin-like 1 and synaptotagmin 1. Our observation that calbindin D28K overexpression increased NF-κB activity points to calbindin D28K acting as an inducer of the NF-κB pathway. This is in line with recent findings indicating that the nuclear export NEMO and its subsequent association with the IKK proteins is inducible by calcium [36]. We also found that overexpression of synaptotagmin 1 markedly diminished TNFα-dependent NF-κB activity, which would otherwise suggest that this protein is a repressor of the NF-κB pathway. We remain skeptical of this idea, however, because when synaptotagmin 1 is overexpressed in HEK-293 cells it localizes almost homogeneously throughout the cell membrane [37 and data not shown]. This may have had the unintended effect of disrupting the activity or structure of the TNF receptor and thus prevented proper activation of the NF-κB pathway upon TNFα addition. It also remains to be seen how synaptotagmin 1 impacts upon NF-κB activity in neurons and also whether or not NEMO is indeed able to localize to the neuronal synapse where synaptotagmin 1 is normally present.

Another of the NEMO interactors, SAG, has been characterized as a cellular antioxidant whose overexpression markedly reduces cell death following stroke or treatment with apoptosis inducers [38], [39]. The E3 ligase activity of SAG is known to promote ubiquitination and subsequent degradation of IκBα, resulting in activation of the NF-κB pathway [25]. It remains unclear how the SAG-NEMO interaction impacts upon this activity, though recent data from our lab suggests that overexpression of SAG can also promote ubiquitination of NEMO (B. Fenner, unpublished results). Recent work has also revealed that SAG is transcriptionally induced under hypoxia by HIF1α [40]. We are currently investigating the impact of SAG on hypoxia-dependent induction of NF-κB.

Cyclin-dependent protein kinase 2, another of the novel NEMO interactors identified here, associates with NF-κB during the G1/S cell cycle transition [24]. We found that CDK2 is required for maximal NF-κB activation in response to TNFα stimulation, though we do not yet know whether this effect is mediated by NEMO, NF-κB or another unidentified intermediate.

SUMOylation of NEMO retains the protein inside the nucleus, and in response to certain cellular stresses NEMO is deSUMOylated, exits the nucleus and promotes activation of the NF-κB pathway [13], [26], [41], [42], [43]. It was therefore of special interest that we identified SENP2, a nuclear-envelope-associated SUMO protease [44], as a NEMO interactor. Previous work indicates that NEMO can be deSUMOylated by SENP1 [14], another nuclear SUMO protease closely related to SENP2, and the activity of this protein may explain why we observed such a modest effect on NF-κB activity following efficient siRNA knockdown of SENP2. What is more intriguing though is why SENP2 associated with NEMO in what would presumably be the absence of SUMOylation during the microarray screen. Current models of deSUMOylation by SENPs do not suggest SUMO-independent recognition of a SUMOylated protein by SENPs [45], [46]. It may therefore be of substantial interest to the field whether SENP2 can indeed deSUMOylate NEMO *in vivo* and how this reaction proceeds in relation to the SUMOylation status of NEMO.

We have identified several new NEMO protein interactors, and have putatively identified over a hundred novel interactors using protein microarrays. We expect that many researchers will be able to use this list to gain new insight into how the NF-κB pathway communicates with other signaling pathways. Future work from our laboratory will focus on more detailed analyses of several of the interactors and how they communicate with NEMO and the NF-κB signaling pathways.

## Materials and Methods

### Preparation of Recombinant NEMO Probe

Human NEMO was expressed as a soluble GST fusion from the pGEX-4T vector [10] in *E. coli* BL21(DE3) and purified using glutathione sepharose (GE Life Sciences) as described previously [10]. For screening of colony macroarrays, the GST-NEMO fusion was used with the GST tag. For screening of protein microarrays, the GST tag was removed following the final GSH-sepharose washing step by adding thrombin (GE Life Sciences) as suggested by the manufacturer, following by size exclusion filtration using a 50 kDa cut-off Microcon filter (Millipore). Approximately 15 µg of NEMO was then biotinylated using an *in vitro* biotinylation kit as suggested by the manufacturer (Invitrogen) with a biotin∶NEMO molar ratio of 9∶1. Biotinylated recombinant NEMO was stored at −80°C.

### Protein Array Screening

ImaGenes UniPex colony macroarrays were screened using a previously described procedure [12]. Version 4.0 of the Invitrogen Protoarray was used for protein microarray screening experiments. After blocking the array in blocking buffer (PBS, 1% BSA, 0.1% Tween 20) for 1 h at 4°C, 10 µg aliquots of biotinylated NEMO or GST control diluted in 120 µl of probing buffer (PBS containing 0.5 mM DTT, 5 mM MgCl_2_, 5% glycerol, 0.05% Triton X-100 and Calbiochem protease inhibitor cocktail) were added to the array and the array covered and incubated at 4°C for a further 1.5 h. Arrays were then washed three times in ice cold probing buffer prior to the addition of streptavidin-alkaline phosphatase (Invitrogen) diluted in probing buffer to a final concentration of 0.25 µg/ml. Arrays were incubated for 30 min on ice, washed three times in probing buffer and then dried for 2–3 h.

Arrays were imaged using a Perkin Elmer Scanarray ExpressHT system and the images analyzed using Invitrogen Prospector version 4 software. Significant interactions were identified based on a Z-score cutoff value of 3.0, with the data obtained from the biotin-GST experiment being subtracted from those of the biotin-NEMO experiment.

### GST Pulldown and Coimmunoprecipitation

GST pulldowns using purified GST-NEMO and transfected HEK-293T cells expressing IKKβ [47], CALB1 (Origene), CDK2-HA [48], SAG (Origene), SENP2-FLAG [49] or SYT1-FLAG (this study) were performed as described previously [12]. For coimmunoprecipitation, HEK293 cells were grown in 6-well plates containing DMEM supplemented with 10% FBS to a confluence of approximately 80%. After rinsing in serum-free DMEM, cells were transfected with 4 µg of pcDNA4/HisMaxA-NEMO [12] and each of the abovementioned plasmids using Lipofectamine 2000 (Invitrogen). At 24 h post-transfection, cells were gently rinsed in ice-cold PBS and scraped into 0.5 ml of lysis buffer (20 mM Tris-HCl, pH 7.5, 137 mM NaCl, 0.5% NP-40, 0.5 mM DTT, 10% glycerol, 2 mM EDTA, Calbiochem protease inhibitor cocktail). The lysate was incubated with end-over-end mixing for 30 min at 4°C and insoluble material pelleted by centrifugation at 10,000×g for 20 min. Supernatants were collected and used for coimmunoprecipitation. A 1 µg aliquot of mouse monoclonal anti-Xpress (Invitrogen) was added to 400 µl (500–750 µg total protein) of the cell lysates and the mixtures incubated with end-over-end mixing for 2 h at 4°C. The resulting immunocomplexes were precipitated by adding 25 µl bed volume of protein A/G agarose (Santa Cruz Biotech) and further incubating with mixing at 4°C for 2 h. The agarose beads were then pelleted at 1,000×g for 3 min and washed three to five times in chilled lysis buffer. Pellets were finally resuspended in 60 µl of 2× Laemmli sample buffer, boiled, and the supernatants collected. Aliquots of 20 µl were used for SDS-PAGE followed by anti-FLAG, anti-Myc, anti-HA or anti-Xpress (Invitrogen) immunoblotting and detection with Dura chemiluminescent substrate (Peirce).

### Mammalian Two-Hybrid Assays

Protein-protein interactions were confirmed by two-hybrid analysis using the CheckMate/Flexi system (Promega) with HEK-293T cells. Two-hybrid complementation plasmids were generated by *Pfu* polymerase-mediated PCR using the abovementioned plasmids as templates with cloning being performed using *Sgf*I and *Pme*I restriction digestion of pFN10A (activation domain) and pFN11A (binding domain). Plasmid sequences were validated by DNA sequencing. Firefly and Renilla luciferase activities were determined using a Dual-Luciferase kit (Promega) and expressed as normalized firefly/Renilla luciferase values.

### NF-κB Reporter Ggene Assays

Transcriptional activation by NF-κB was monitored using a secreted alkaline phosphatase (SEAP) reporter plasmid, pNF-κB-SEAP, which contains four κB consensus sequences upstream of SEAP [50]. HEK-293T cells were seeded in 24-well plates and grown overnight in antibiotic-free DMEM containing 10% FCS to approximately 60% confluence. Cells were then transfected with 400 ng of reporter plasmid and 1600 ng of the different expression plasmids using Lipofectamine 2000 (Invitrogen) and serum-free DMEM as recommended by the manufacturer. Medium was replaced with serum-containing medium at 4–6 h post-transfection. At this point, cells were treated with TNFα (100 ng/ml) or left untreated for a further 20 h. Culture supernatants were then collected and heat-treated at 65°C for 5 min to inactivate endogenous alkaline phosphatase. Cells were washed in PBS and lysed directly using 50 µl of Laemmli sample buffer for protein expression analysis by immunoblotting. SEAP was measured in 96-well plates by combining 10 µl of supernatant with 190 µl of Attophos substrate (Roche) and incubating plates at room temperature for 12 h prior to reading fluorescence with a BioTek Synergy HT plate reader (Ex_485_/Em_528_).

### RNA Interference

HEK-293T cells were transfected with 400 ng of the NF-κB reporter plasmid as described above, along with 20 pmol of siRNAs directed against CDK2 (Ambion Silencer siRNA S204), SAG (Ambion Silencer siRNA S18473), SENP2 (Ambion Silencer siRNA S34009) or a nonspecific control (Ambion Silencer control siRNA). Pre-incubation of the siRNA and plasmid DNA with the Lipofectamine 2000 was reduced to 5 min to reduce siRNA degradation. Cells were incubated with the Lipofectamine 2000 complexes for 5 hours prior to replacement of the medium with DMEM containing 10% FBS and further incubated for 48 h. The medium was then replaced and the cells treated with TNFα for a further 24 h, at which time the SEAP reporter activity was determined as described above and cellular RNA prepared using a Qiagen RNeasy kit and Invitrogen RNase-free DNase.

### Quantitative PCR

Amounts of CDK2, SAG, SENP2 and 18s rRNA were quantitated by RT-qPCR using a LightCycler RNA Master SYBR Green I kit (Roche) with gene-specific primers CDK2-FWD1 (5′-GGAGAACTTCCAAAAGGTGG-3′) and CDK2-REV1 (5′-TCTCGGATGGCAGTACTGGG-3′), SAG-FWD1 (5′-CGACGTGGAAGACGGAGAGGAA-3′) and SAG-REV1 (5′-TGCAGATGGCGCACGTATCG-3′), SENP2 FWD1 (5′-ATGTACAGATGGCTGGTTAG-3′) and SENP2 REV1 (5′-TGGTCTTTTGGCTGGTATTT-3′), or 18S FWD1 (5′-GTAACCCGTTGAACCCCATT-3′) and 18S REV1 (5′-CCATCCAATCGGTAGTAGCG-3′). Amounts of each mRNA were calculated relative to the 18S rRNA control and values expressed as percentages of the control siRNA sample for each siRNA knockdown.
